# Early Detection of Poor Adherers to Statins: Applying Individualized Surveillance to Pay for Performance

**DOI:** 10.1371/journal.pone.0079611

**Published:** 2013-11-04

**Authors:** Andrew J. Zimolzak, Claire M. Spettell, Joaquim Fernandes, Vincent A. Fusaro, Nathan P. Palmer, Suchi Saria, Isaac S. Kohane, Magdalena A. Jonikas, Kenneth D. Mandl

**Affiliations:** 1 Children’s Hospital Informatics Program at Harvard-Massachusetts Institute of Technology Health Sciences and Technology, Boston Children’s Hospital, Boston, Massachusetts, United States of America; 2 Aetna, Blue Bell, Pennsylvania, , United States of America; 3 Center for Biomedical Informatics, Harvard Medical School, Boston, Massachusetts, United States of America; 4 Division of Health Sciences & Informatics, Johns Hopkins University, Baltimore, Maryland, United States of America; 5 Department of Pediatrics, Harvard Medical School, Boston, Massachusetts, United States of America; University of Milan, Italy

## Abstract

**Background:**

Medication nonadherence costs $300 billion annually in the US. Medicare Advantage plans have a financial incentive to increase medication adherence among members because the Centers for Medicare and Medicaid Services (CMS) now awards substantive bonus payments to such plans, based in part on population adherence to chronic medications. We sought to build an individualized surveillance model that detects early which beneficiaries will fall below the CMS adherence threshold.

**Methods:**

This was a retrospective study of over 210,000 beneficiaries initiating statins, in a database of private insurance claims, from 2008-2011. A logistic regression model was constructed to use statin adherence from initiation to day 90 to predict beneficiaries who would not meet the CMS measure of proportion of days covered 0.8 or above, from day 91 to 365. The model controlled for 15 additional characteristics. In a sensitivity analysis, we varied the number of days of adherence data used for prediction.

**Results:**

Lower adherence in the first 90 days was the strongest predictor of one-year nonadherence, with an odds ratio of 25.0 (95% confidence interval 23.7-26.5) for poor adherence at one year. The model had an area under the receiver operating characteristic curve of 0.80. Sensitivity analysis revealed that predictions of comparable accuracy could be made only 40 days after statin initiation. When members with 30-day supplies for their first statin fill had predictions made at 40 days, and members with 90-day supplies for their first fill had predictions made at 100 days, poor adherence could be predicted with 86% positive predictive value.

**Conclusions:**

To preserve their Medicare Star ratings, plan managers should identify or develop effective programs to improve adherence. An individualized surveillance approach can be used to target members who would most benefit, recognizing the tradeoff between improved model performance over time and the advantage of earlier detection.

## Introduction

Poor medication adherence costs the U.S. healthcare system up to nearly $300 billion[1-3] each year. Patients who adhere to prescribed medication have fewer hospitalizations, lower costs[4], and lower mortality[5], as compared to their non-adherent counterparts. Nonadherence is common; in studies of statin adherence, only around 50% of subjects remain fully adherent 6 months after initiation[6,7]. However, interventions to improve adherence are often cost- and time-intensive[8,9]. 

In 2012, as required by the Patient Protection and Affordable Care Act, the Centers for Medicare and Medicaid Services (CMS) began using star ratings to award substantive bonus payments to privately-run Medicare Advantage and stand-alone Medicare prescription drug plans[8,10,11]. Higher star ratings are also associated with higher plan enrollment[12]. An important component of assigning these ratings is the proportion of plan beneficiaries who achieve a proportion of days covered (PDC) of 0.8 or above[11], for three classes of medications: statins, renin angiotensin system antagonists, and oral hypoglycemic agents. The adherence measures for these classes are obtained from prescription fulfillment data and are measured over a one-year period. We sought to develop a predictive model of poor adherence to statins so that plan managers, early in the year, can efficiently implement and target programs to the right patients.

## Methods

### Design

This was a retrospective database study using de-identified medical and pharmacy claims, on all Aetna commercial members with at least one year of continuous medical and pharmacy coverage from 2008-2011. Patients were included if they received a statin prescription and met criteria for dyslipidemia, where dyslipidemia was defined as: 2 claims with a diagnosis of lipid disorder (International Classification of Diseases, Ninth Revision (ICD-9) diagnosis code of 272.x) unrelated to a laboratory claim, or low-density lipoprotein cholesterol >130 mg/dL (to convert to millimoles per liter, multiply by 0.0259), or total cholesterol >200 mg/dL, or HDL cholesterol <40 mg/dL, or triglycerides >150 mg/dL (to convert to millimoles per liter, multiply by 0.0113), or a claim with a Current Procedural Terminology Category II code for a lipid disorder (3049F, 3050F, 4013F, 0556F, or 4002F). Raw data are available upon request.

Members with unknown gender or with ages <0 or >100 years were excluded, as were members who received high fill quantities (>100 days) likely to be in error or not reflective of true days covered. The members (<0.1% of total) taking lovastatin were excluded from final analysis because this very low prevalence category destabilized the model. Members were excluded if they had less than 90 days of continuous eligibility after statin initiation. Finally, to provide a “wash out” period that would separate those truly initiating statins from those merely continuing statins after switching plans, members were excluded if the time from first eligibility to first statin prescription was less than 180 days. The data were divided into training and validation subsets (two thirds and one third of members, respectively).

### Ethics statement

The study was reviewed and approved by the Committee on Human Studies of Harvard Medical School. Consent was waived by the Committee, which identified the study as not involving human subjects per Federal regulations.

### Adherence measurement

For each member, we calculated PDC for days 91-365 (PDC91-365), dichotomized at the 0.8 level, to be used as the outcome variable. This measure calculates adherence in a period that does not overlap with the early detection period. Day 1 (index date) is defined as the fill date for the first statin prescription for that member. All PDC calculations were performed according to methods described by CMS.[11] Specifically, PDC was defined as the number of days that the member was covered by at least one statin, divided by the number of days in the measurement period. PDC over 100% (when the member fills more than expected) is expressed as 100%. Days of overlap between two fills counted toward PDC, but only if the two fills were for the same statin. If prescriptions in days 1-90 had days of supply that extended into the days 91-365 period, those days of supply contributed toward PDC91-365. The days covered and the duration of the measurement period were adjusted for inpatient stays according to the CMS methods (“drug coverage during the inpatient stay is shifted to subsequent days of no supply,” and “the days of inpatient stay are deleted from the measurement period”)[11]. For members with less than one year of eligibility after the index date, PDC91-365 could not be calculated.

### Baseline variables used to predict adherence

**Figure 1:**
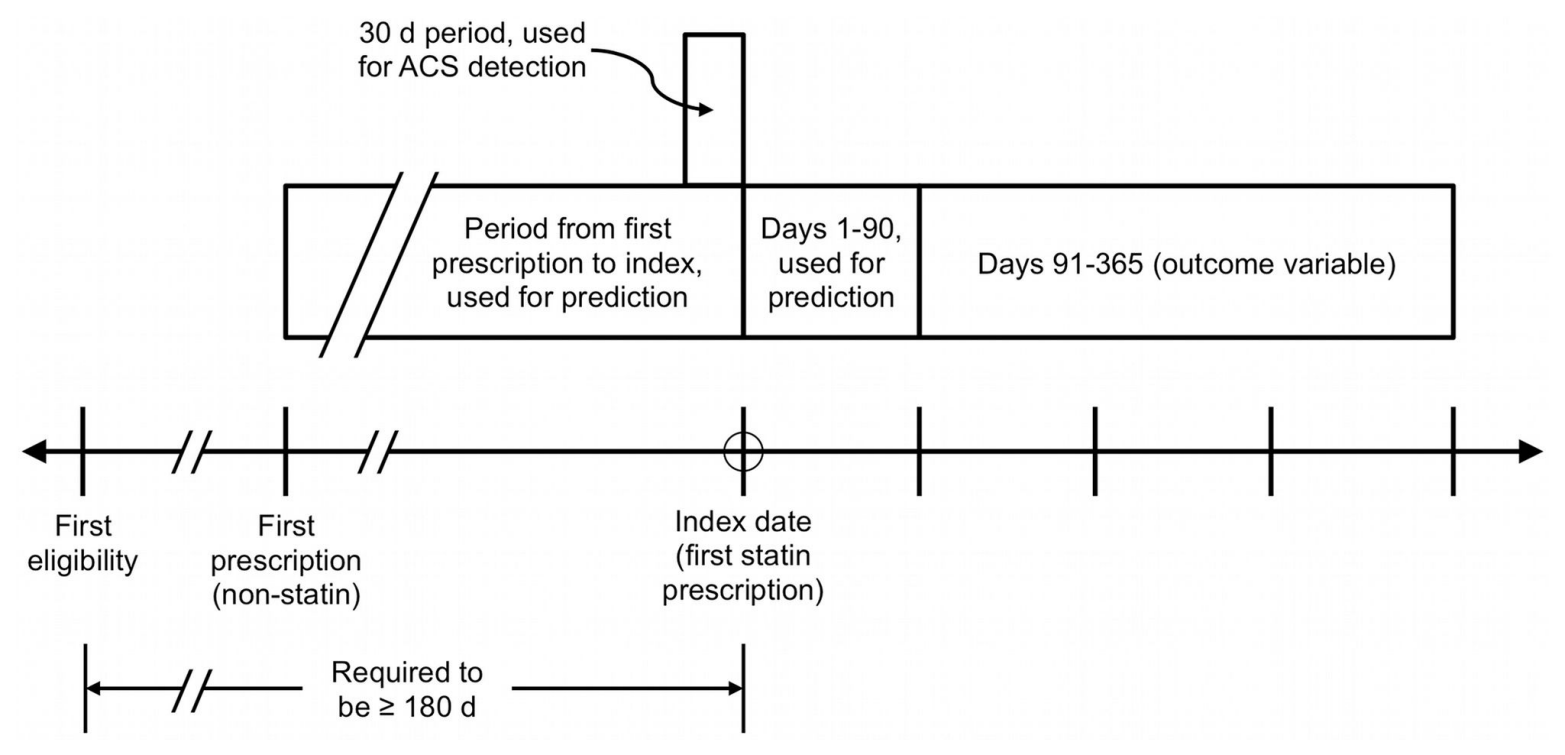
Timeline showing periods used to calculate adherence measures and baseline variables. Outcome variable is proportion of days covered (PDC) by statin for days 91-365, respectively. Three baseline variables are calculated from the first non-statin prescription to the index date. Presence of ACS (acute coronary syndrome) as a baseline variable was determined in the 30 days prior to statin initiation. Nine baseline variables including early PDC are calculated from statin prescriptions in days 1-90. Time from eligibility to initiation is required to be 180 days (in both models) in order to include those truly initiating statins, and not those merely continuing statins after switching insurance plans.

The primary independent measure was PDC for days 1-90, analyzed as a continuous variable (PDC1-90). Model building was similar to a prior analysis, on a different population, predicting medication possession ratio[13]. We considered 15 additional variables focusing on those previously shown to be associated with adherence[1,6,7,14]. Age, gender, and statin name were available from claims data. For statin name, simvastatin was designated as the comparator for all other categories. We calculated the following variables from the first 90 days only after the index date (Figure 1): proportion of statin fills at retail pharmacies, proportion of formulary statin fills, proportion of generic statin fills, average days supply per statin prescription, average statin reimbursement per prescription, average statin copayment per prescription, and average statin dose per prescription. Finally, we calculated the following variables to describe the period from the first non-statin prescription to the index date (Figure 1): average pills per day, average non-statin reimbursement per day, average non-statin copayment per day, presence of acute coronary syndrome within 30 days prior to index date (defined as hospitalization for unstable angina or myocardial infarction, ICD-9 codes 411.x or 410.x), and time from first eligibility to index date.

### Analyses

Using logistic regression, bivariate associations were explored between baseline variables and PDC91-365 (dichotomized at 0.8). For continuous baseline variables, units for the odds ratios were set equal to 2 standard deviations of the variable. This method allows comparison of continuous variables’ impact on model discrimination[15]. A multivariable logistic regression model was developed on the training set, using continuous PDC1-90 and the 15 additional variables to predict probability of poor adherence (PDC91-365 dichotomized at 0.8). All baseline variables were included, and no further selection of variables was performed.

The trained model was applied to the validation set, and a receiver operating characteristic (ROC) curve was plotted. Odds ratios with 95% confidence intervals were determined for each variable. For continuous variables, units for the odds ratios were set equal to 2 standard deviations of the variable. We applied the model to all beneficiaries in the validation set. However, those who left the plan before year’s end had censored data. For those with censored data, model predictions can be made but not classified as true or false; therefore members with censored data do not contribute to sensitivity and specificity calculations. However, we report numbers of members with censored data because, from the plan manager’s perspective, resources would be devoted toward those at risk for nonadherence, and it would not be known in advance which members would leave the plan before one year.

Our primary multivariable model requires 90 days of data to predict future nonadherence, but in practice an insurance plan manager may be willing to sacrifice model performance for a prediction sooner than 90 days. Thus, we performed a sensitivity analysis, varying the date of prediction from 0 to 270 days, and examining impact on prediction AUC. We stratified this analysis by the days’ supply of the first statin fill (≤30 days versus >30 days). We inspected the performance vs. time curves for both subsets and also calculated performance characteristics that would result if predictions were made at different time points for the two subsets (as opposed to our single 90-day time point used in the primary model). All analyses were performed using SAS statistical software, version 9.3 (SAS Institute Inc, Cary, NC).

## Results

**Figure 2:**
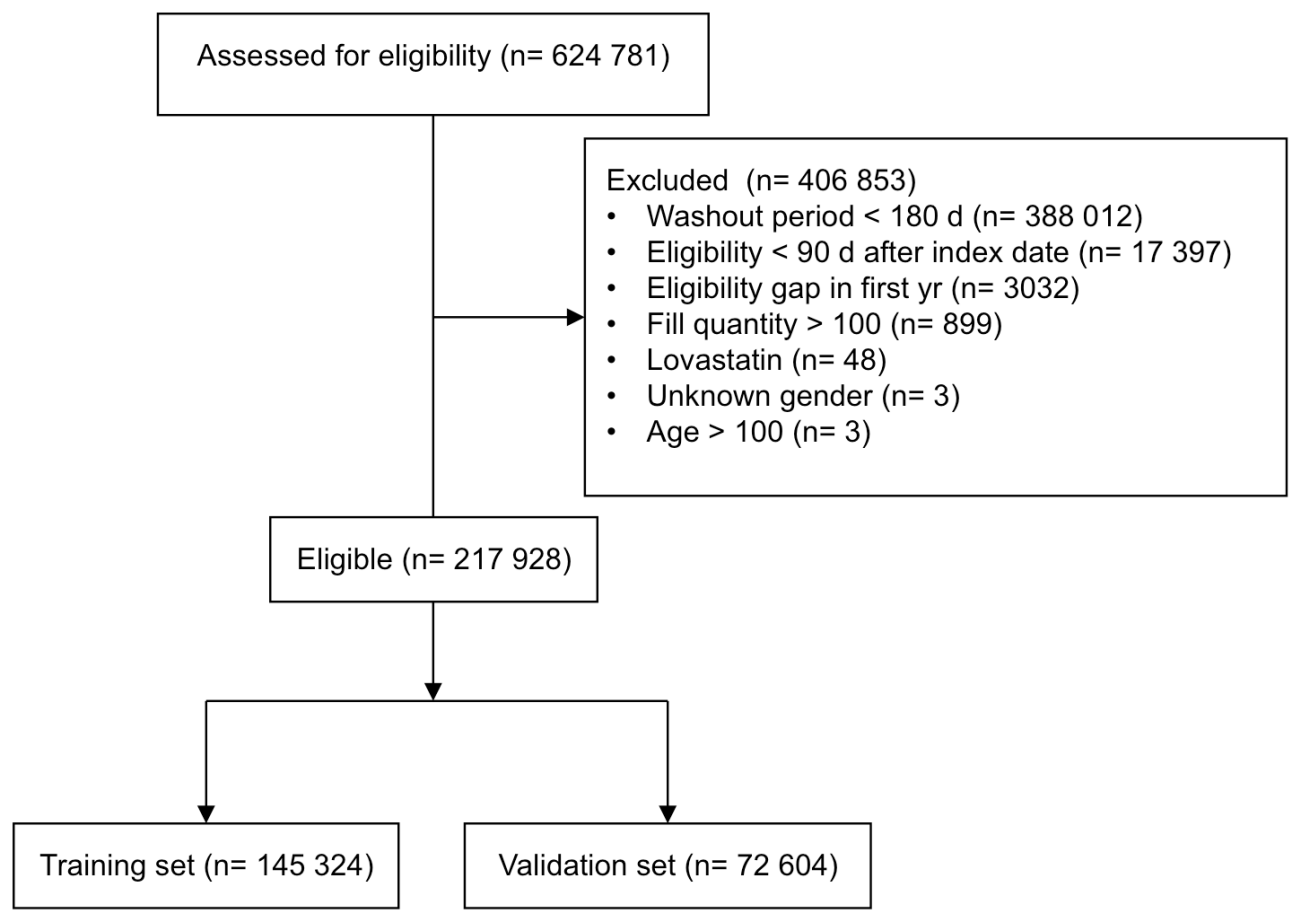
Study flow diagram showing exclusion criteria. The sum of members who met each individual exclusion criterion does not equal the total number excluded because one member can meet more than one exclusion criterion.

A total of 624,781 members met inclusion criteria. After exclusion criteria were applied, 217,928 remained (Figure 2). Table 1 shows baseline characteristics of the members.

**Table 1:** Baseline characteristics of the study population before and after exclusion criteria^a^.

**Characteristic**	**All members (n = 624781)**	**Selected cohort (n = 217928)**
Age, median (IQR), y	56 (49-62)	54 (47-61)
Sex		
Women	264541 (42.3)	98840 (45.4)
Men	360237 (57.7)	119088 (54.6)
PDC, days 1-90, median (IQR)	0.96 (0.67-1.00)	0.87 (0.59-1.00)
PDC, days 1-365, median (IQR)	0.76 (0.41-0.95)	0.58 (0.25-0.89)
< 0.8	268457 (43.0)	100879 (46.3)
≥ 0.8	240277 (38.5)	51223 (23.5)
Unknown (censored)	116047 (18.6)	65826 (30.2)
PDC, days 91-365, median (IQR)	0.73 (0.33-0.94)	0.51 (0.11-0.87)
< 0.8	285008 (45.6)	104750 (48.1)
≥ 0.8	223726 (35.8)	47352 (21.7)
Unknown (censored)	116047 (18.6)	65826 (30.2)
Medication		
Simvastatin	318408 (51.0)	126464 (58.0)
Atorvastatin	159434 (25.5)	33777 (15.5)
Rosuvastatin	127800 (20.5)	52466 (24.1)
Fluvastatin	6528 (1.0)	700 (0.3)
Pravastatin	1176 (0.2)	323 (0.1)
Lovastatin	48 (0.0)	0
Multiple	11387 (1.8)	4198 (1.9)
Source of majority of statins, days 1-90		
Mail order	122852 (19.7)	24669 (11.3)
Retail	501929 (80.3)	193259 (88.7)
Formulary status of majority of statins, days 1-90		
Formulary	457886 (73.3)	182061 (83.5)
Nonformulary	166895 (26.7)	35867 (16.5)
Generic status of majority of statins, days 1-90		
Generic	319374 (51.1)	127235 (58.4)
Brand-name	305407 (48.9)	90693 (41.6)
ACS in 30 days prior to statin initiation	6674 (1.1)	4340 (2.0)
Average supply per statin fill, days 1-90, median (IQR), days	30 (30-60)	30 (30-30)
First fill ≤ 30 d	468354 (75.0)	182989 (84.0)
First fill > 30 d	156427 (25.0)	34939 (16.0)
Average statin payment, days 1-90, median (IQR), $	47.21 (3.08-94.50)	11.73 (2.35-85.14)
Average statin copayment, days 1-90, median (IQR), $	20 (10-40)	15 (10-30)
Average statin dose, days 1-90, median (IQR), mg	20 (10-40)	20 (10-40)
Time from eligibility to statin initiation, median (IQR), days	82 (14-355)	446 (295-679)
Time from statin initiation to end of eligibility, median (IQR), days	734 (443-1129)	541 (317-820)
Pills per day, first prescription ever to statin initiation, median (IQR)	0.00 (0.00-1.26)	0.40 (0.00-1.83)
Payment per day, first prescription ever to statin initiation, median (IQR), $	0.00 (0.00-1.49)	0.16 (0.00-2.17)
Copayment per day, first prescription ever to statin initiation, median (IQR), $	0.00 (0.00-0.98)	0.25 (0.00-1.03)

^a^Values are presented as number (percentage) unless otherwise indicated. Abbreviations: ACS, acute coronary syndrome; IQR, interquartile range; PDC, proportion of days covered.

Table 2 shows the pairwise associations between baseline variables and PDC91-365. Early PDC had the strongest effect of all variables (odds ratio for poor adherence 19.9 per 2 standard deviation decrease in early PDC, 95% confidence interval 19.0-20.9).

**Table 2:** Bivariate and multivariable associations between baseline variables and outcome (poor adherence)^a^.

**Variable**	**Odds ratio (95% CI)**	**P value**	**Adjusted odds ratio (95% CI)**	**P value**
PDC days 1-90	0.04 (0.04-0.05)	<.001	0.04 (0.04-0.04)	<.001
Age	0.54 (0.53-0.55)	<.001	0.64 (0.62-0.66)	<.001
Mail order source of majority of statins, days 1-90	0.42 (0.40-0.44)	<.001	0.67 (0.63-0.71)	<.001
Pills per day, prior to statin initiation	0.64 (0.62-0.66)	<.001	0.78 (0.75-0.81)	<.001
Brand-name status of majority of statins, days 1-90	1.08 (1.05-1.11)	<.001	0.86 (0.69-1.05)	0.140
Average reimbursed amount per day, prior to statin initiation	0.71 (0.69-0.74)	<.001	0.97 (0.93-1.00)	0.088
Average copayment per day, prior to statin initiation	0.75 (0.71-0.79)	<.001	0.98 (0.96-1.01)	0.265
Average statin dose, first 90 days of statin era	0.97 (0.95-1.00)	0.020	1.09 (1.06-1.13)	<.001
Days from eligibility to statin initiation	1.05 (1.02-1.08)	0.004	1.10 (1.06-1.14)	<.001
Average reimbursed amount, first 90 days of statin era	0.71 (0.69-0.73)	<.001	1.10 (1.04-1.16)	0.001
Nonformulary status of majority of statins, days 1-90	1.09 (1.06-1.13)	<.001	1.09 (0.94-1.27)	0.238
Average copayment, first 90 days of statin era	0.97 (0.94-0.99)	0.011	1.12 (1.08-1.16)	<.001
Female sex	1.12 (1.09-1.15)	<.001	1.18 (1.14-1.21)	<.001
No ACS within 30 prior days	2.27 (2.08-2.48)	<.001	1.42 (1.28-1.58)	<.001
Average days supply, first 90 days of statin era	0.58 (0.57-0.60)	<.001	1.63 (1.56-1.70)	<.001
Medication (Simvastatin is the comparator for all categories.)				
Atorvastatin	1.13 (1.09-1.18)	0.479	1.07 (0.88-1.29)	0.129
Rosuvastatin	1.06 (1.03-1.10)	0.405	1.08 (0.87-1.33)	0.212
Pravastatin	1.84 (1.23-2.74)	0.003	2.40 (1.52-3.82)	<.001
Fluvastatin	1.18 (0.94-1.49)	0.486	1.26 (0.90-1.76)	0.552
Multiple	0.68 (0.62-0.74)	<.001	0.73 (0.62-0.86)	<.001

^a^Units for continuous variable odds ratios: PDC, 0.5; age, 20 years; pills per day, 5; reimbursement per day, $20; copayment per day, $6; average statin days supply, 40; time from eligibility to index, 520 days; average statin reimbursement, $140, average statin copayment, $50, average statin dose, 30 mg. Abbreviations: PDC, proportion of days covered; ACS, acute coronary syndrome.

In multivariable analysis, the model had an AUC of 0.80 for the training set and validation set both. Adjusted odds ratios and 95% confidence intervals for all variables are shown in Table 2. Early PDC had the strongest effect of all variables in the model. Specifically, patients with a lower PDC at 90 days had an odds ratio of 25.0 (95% confidence interval 23.7-26.5) for poor adherence from days 91 to 365. The positive predictive value for poor adherence is 87.7%, and negative predictive value is 53.4%. The model can predict the CMS definition of statin nonadherence with 69.5% sensitivity and 78.2% specificity. For every 1000 beneficiaries, the model predicts poor adherence for 562. Of those, 335 are true positives, 47 are false positives and 180 are censored (eligibility ends within a year of statin initiation). The model predicts good adherence for 438 beneficiaries, of whom 168 are true negatives, 147 are false negatives, and 123 are censored.

**Figure 3:**
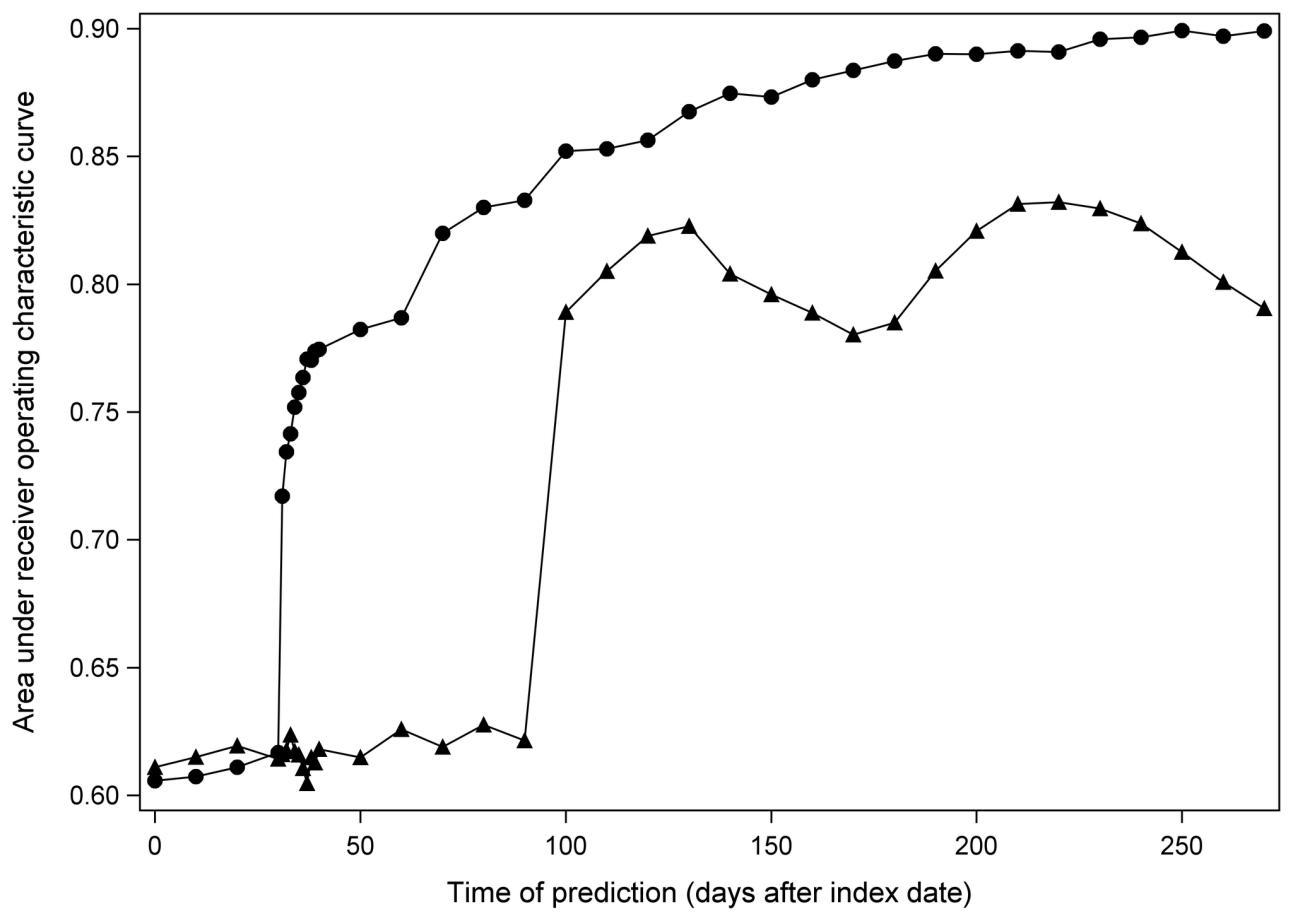
Model performance versus time of prediction. Separate curves are presented for members whose first statin fill was for 30 days or less (circles), and for members whose first statin fill was for more than 30 days (triangles). For the ≤30 day subset, performance improves sharply between days 30 and 31, and it improves steadily thereafter. However, for the >30 day subset, (most of whom had 90-day fills,) performance improves sharply only after day 90. Each Y coordinate expresses the performance of a model that uses prescription fills from days 1 to X to predict adherence from days X to 365.

In the sensitivity analysis, models did not perform well if they were based on less than 30 days of early adherence monitoring (AUCs near 0.6). Model performance increases sharply from days 30 to 31 (from AUC 0.62 to 0.71) for those members whose first prescription was for a 30-day supply or less, and it improved steadily thereafter. For those members whose first prescription was for more than 30 days, (most of whom received 90-day supplies,) AUC did not increase until after day 90 (Figure 3). When members with 30-day supplies for their first statin fill had predictions made at 40 days, and members with 90-day supplies for their first fill had predictions made at 100 days, poor adherence could be predicted with 86% positive predictive value, 50% negative predictive value, 69% sensitivity, and 74% specificity.

## Discussion

Early PDC is a strong indicator of future nonadherence, and, in fact stronger than variables identified in prior studies[8] which have shown relatively low predictive power of insurance claims, with AUCs under 0.64[14]. We show, however, that while claims data have limited value prior to initiation of the medication, they contain important information after several weeks of filling behavior, as they become an indicator of personal behavior[16].

Because CMS now uses adherence to award bonus payments, adherence is tied to direct financial incentives as well as incentives to prevent morbidity and mortality. We show that the CMS metric of PDC can be predicted far in advance; therefore plan managers could apply an individualized surveillance model to target adherence improvement programs to their Medicare Advantage members who are at risk for nonadherence and who are likely to benefit from such programs.

Prediction of PDC improves with time. Our primary model, which uses 90 days of adherence data, performs well, but sensitivity analysis demonstrates the utility of early monitoring as early as day 31. Little improvement in model performance is gained from monitoring adherence before the first prescription fill is expected to run out. A plan manager can account for the tradeoff between early detection of poor adherers and accuracy, based on the cost and characteristics of the intervention. 

A limitation of this study, in common with much claims-based adherence research, and in fact with the very PDC metric used by CMS, is that it treats medication fills as essentially the same as adherence, which is a simplified conception[8]. Our model does not address self-report, pill counts, or other measures of adherence, and further research should use these measures as outcome variables, either singly or in combination. Similarly, all purely claims-based adherence research, including this study, cannot address underlying reasons for treatment discontinuations, including discontinuations that are recommended by the prescriber. Adherence is commonly defined as “the extent to which patients take medications as prescribed by their health care providers,”[1] and this cannot strictly be assessed with prescription claims data alone. Second, there is only slim evidence that the 0.8 PDC threshold is clinically meaningful[8,17]. Third, it may be difficult to separate any true effect of 90-day prescription supplies on adherence from the effect of days supply on the PDC calculation. Further study is particularly needed to determine the effect of days supply on pill taking, not just on metrics of prescription filling. Finally, our source population is commercial insurance members who were initiating statins; we did not study the Medicare population or those who were already taking statins when they entered our dataset. These populations are important for the proposed application of our model, and its performance should be confirmed in these populations.

To further increase accuracy of detection, the use of additional data sources, including electronic medical record information and patient-reported adherence, should be explored.
